# Finerenone in Patients with Nondiabetic Chronic Kidney Disease—A Retrospective Study

**DOI:** 10.3390/biomedicines13102519

**Published:** 2025-10-15

**Authors:** Rehab B. Albakr, Fadel AlRowaie, Ibrahim A. Sandokji, Yazid A. Alhadlg, Khalid Almatham, Abdulaziz B. Albacker

**Affiliations:** 1 Department of Medicine, Division of Nephrology, College of Medicine, King Saud University, Riyadh 11472, Saudi Arabia; 2 Department of Medicine, Division of Nephrology, King Fahad Medical City, Riyadh 12231, Saudi Arabia; falrowaie@kfmc.med.sa (F.A.); yazidalhadlg18@gmail.com (Y.A.A.); kalmatham@gmail.com (K.A.); 3 College of Medicine, AlFaisal University, Riyadh 11533, Saudi Arabia; 4 Pediatric Department, College of Medicine, Taibah University, Madinah 42353, Saudi Arabia; isandokji@gmail.com; 5 Department of Internal Medicine, College of Medicine, King Saud University, Riyadh 11472, Saudi Arabia; aziz.backer32112@gmail.com

**Keywords:** nondiabetic CKD, finerenone, nonsteroidal mineralocorticoid receptor, eGFR, UACR, hypotension, hyperkalemia

## Abstract

**Background & Objectives:** Data on the efficacy and adverse effects of finerenone in patients with nondiabetic chronic kidney disease (CKD) are limited, particularly regarding ethnic diversity. This study aimed to evaluate the outcomes of finerenone in patients with nondiabetic CKD previously treated with standard therapies and investigate associated adverse effects, including hyperkalemia and hypotension. **Methods:** This is a retrospective exploratory study. It is a single-center study including patients with nondiabetic CKD who visited King Fahad Medical City in Riyadh, Saudi Arabia. The primary exposure was finerenone treatment, assessing its effects on albuminuria, kidney function, and blood pressure (BP), following prior use of renin–angiotensin–aldosterone system and sodium–glucose transport protein 2 inhibitors. The measured outcomes were the urine albumin-to-creatinine ratio (UACR) and estimated glomerular filtration rate (eGFR). The UACR (primary endpoint) was calculated as the mean of two morning spot urine samples collected consecutively 1 day apart. During each 4-week treatment period, secondary endpoints included changes in UACR, as determined by a 24 h urine sample, BP, and eGFR. The Wilcoxon signed-rank test was used to compare changes in continuous variables before and after therapy initiation. Statistical significance was set at *p* < 0.05. **Results:** This study included 16 patients with nondiabetic CKD (median age, 38.5 years [range, 35–50 years]; 56.3% male). The baseline eGFR was 66 mL/min/1.73 m^2^ (47–82.5), with a UACR of 90.0 mg/g (58.8–132.5). No hyperkalemia was observed (potassium level, 4 mmol/L [3.8–4.4]). However, significant reductions in systolic and diastolic BPs were observed. Albuminuria improved significantly: the UACR decreased from 90.0 to 39.3 mg/g (*p* = 0.04). No adverse events, including hyperkalemia or hypotension, were reported. **Conclusions:** Finerenone showed promise in reducing albuminuria and blood pressure among patients with nondiabetic chronic kidney disease, with no significant adverse effects reported. These findings suggest potential benefits for this patient population, warranting further investigation.

## 1. Introduction

Mineralocorticoid receptor antagonists (MRAs) have shown promise in the treatment of cardiac insufficiency, resistant hypertension, and various kidney disorders. Finerenone, a selective nonsteroidal MRA, exhibits high potency with reduced adverse effects. It has been associated with improved renal and cardiovascular outcomes for high-risk individuals suffering from chronic kidney disease (CKD). By blocking the effects of mineralocorticoids, finerenone decreases the risk of worsening renal function and cardiovascular incidents in patients with CKD linked to type 2 diabetes mellitus (T2DM). However, its effectiveness and safety in individuals with nondiabetic CKD still require comprehensive validation [1,2]. Although there are distinctions between selective and non-selective MRAs, finerenone has demonstrated superior results regarding reductions in albuminuria and BNP levels without causing significant increases in potassium levels [3].

Finerenone is characterized as a nonsteroidal mineralocorticoid that possesses enhanced pharmacokinetic and pharmacodynamic properties. It is favored over earlier medications like spironolactone and eplerenone due to its lower propensity for cardiorenal complications and hyperkalemia [4,5].

A double-blind study involving 5734 patients with CKD and T2DM divided participants into two groups: one receiving a placebo and the other receiving finerenone. Results indicated that finerenone significantly lowers the risk of progression of chronic renal disease as well as cardiovascular issues among these patients [6]. Another randomized controlled double-blind phase II-b trial conducted in Japan compared finerenone to eplerenone, administering either treatment for 90 days. This trial suggested good tolerance for finerenone among Japanese participants; however, due to the limited sample size, further research is necessary to ascertain its efficacy across larger populations [7].

Finerenone holds significant potential to transform therapeutic approaches for individuals with CKD. A recent study involved 20 patients with nondiabetic CKD who were treated with either finerenone or dapagliflozin over four weeks, followed by a combination therapy for another four weeks. The concurrent use of finerenone alongside dapagliflozin exhibited a favorable safety profile along with a notable decrease in albuminuria [8].

Currently, data on the efficacy of finerenone in patients with nondiabetic chronic kidney disease (CKD) are limited. This highlights a significant knowledge gap, as understanding the drug’s effects across diverse populations is crucial for optimizing treatment strategies. Further studies involving multiple ethnicities are essential to confirm finerenone’s efficacy in improving the prognosis of nondiabetic CKD and to identify potential adverse effects associated with its use in different demographic groups.

The Saudi population presents a unique opportunity for this research, as it reflects a distinct ethnic background that contributes to the global diversity of CKD experiences. Investigating the outcomes of finerenone in this cohort is particularly relevant, given the high prevalence of nondiabetic CKD in the region and the potential for genetic, environmental, and lifestyle factors to influence treatment responses.

This exploratory study aimed to evaluate the effectiveness of finerenone in patients with nondiabetic CKD who had previously been treated with standard therapeutic regimens, including renin–angiotensin–aldosterone system (RAAS) inhibitors and sodium–glucose co-transporter 2 (SGLT2) inhibitors, for managing albuminuria and estimated glomerular filtration rate (eGFR). Additionally, we investigated the adverse effects associated with finerenone, such as hyperkalemia and hypotension. The findings from this study will provide valuable insights into the use of finerenone in non diabetic chronic kidney disease and may serve as a starting point for future prospective large studies.

## 2. Materials and Methods

### 2.1. Study Design

This was a retrospective, single-center study involving patients who visited the outpatient nephrology clinics of King Fahad Medical City in Riyadh, Saudi Arabia. Only individuals with nondiabetic CKD who are on a maximum tolerated dose of RAAS and SGLT2, or not tolerating any of them, and have residual proteinuria for which they started on finerenone were included in the study, and those with a prior diagnosis of diabetes mellitus were excluded.

### 2.2. Albuminuria Measurement

Samples were collected during the visit. Two days before these appointments, patients provided one 24 h urine sample and two morning spot urine samples at home. The mean urine albumin-to-creatinine ratio (UACR) derived from the two-morning spot urine samples was used to minimize variability.

### 2.3. Glomerular Filtration Rate Estimation

The clearance of technetium-99m-DTPA from plasma was used to calculate the measured GFR (mGFR). Four samples were collected from participants with an eGFR of >40 mL/min/1.73 m^2^ at 20 min intervals, 3–4 h after injection. Five samples were collected from participants with an eGFR of ≤40 mL/min/1.73 m^2^ at 30 min intervals, 3–5 h after injection. Blood pressure (BP) was measured while the participants were seated, using a calibrated sphygmomanometer (Microlife WatchBP Office, Microlife AG, Widnau, Switzerland), after a minimum of 5 min repose. Six automated readings were obtained at 1 min intervals, and the average of the five most recent readings was calculated.

### 2.4. Physical Examination

Each patient underwent a physical examination, during which their weight and height were measured and recorded.

### 2.5. End Points

The primary outcome measure was the change in UACR among all participants during the 4-week treatment period, along with changes in BP and eGFR. The safety endpoints were adverse events reported by the investigators.

### 2.6. Statistical Analysis

We summarize and present the characteristics of the study participants, including baseline demographics and clinical and laboratory variables. Categorical variables, such as sex, are presented as percentages, while continuous variables, such as age, are presented as medians with interquartile ranges. The Wilcoxon signed-rank test was used to compare changes in continuous variables, such as BP, before and after therapy initiation. An alpha level of 0.05 was used as the threshold for statistical significance. Data analysis and visualization were performed using Stata version 15.1 and GraphPad Prism version 10.0, respectively.

## 3. Results

Sixteen patients with nondiabetic CKD were included in this study. The median age was 38.5 years (range, 35–50 years), and 56.3% of the patients were male. The median body mass index was 29.0 kg/m^2^ (27.2–30.0). Table 1 presents the baseline characteristics of the study participants. Among the patients, 50% had IgA nephropathy, 18.8% had focal segmental glomerulosclerosis (FSGS), and 31.3% had other etiologies. The baseline systolic and diastolic BPs were 131.5 mmHg (122.5–139.5) and 79 mmHg (74.5–85), respectively. The baseline estimated eGFR was 66 mL/min/1.73 m^2^ (47–82.5), with a UACR of 90.0 mg/g (58.8–132.5). No hyperkalemia was observed, with a potassium level of 4 mmol/L (3.8–4.4). Table 1 also shows the use of various medications across the sample population. RAAS inhibitors were used by all participants (100%). SGLT2 inhibitors and statins were administered to 93.8% and 81.3% of the patients, respectively. In contrast, diuretics and potassium binders were administered to only 18.8% and 12.5% of the patients, respectively. Among immunosuppressants, mycophenolate mofetil was the most commonly prescribed (37.5%), followed by prednisolone (12.5%) and hydroxychloroquine (6.3%).

**Figure 1:**
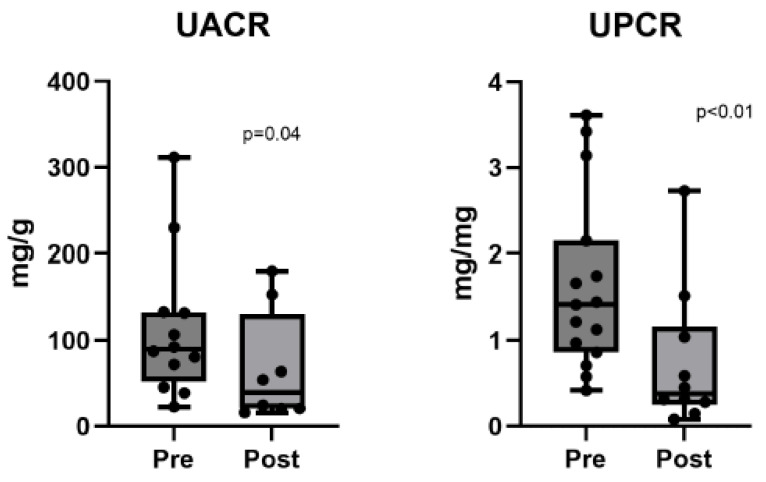
Differences in UPCR (n = 10) and UACR (n = 8) pre- and post-finerenone treatment in patients with nondiabetic chronic kidney disease. UPCR, urine protein-to-creatinine ratio; UACR, urine albumin-to-creatinine ratio.

**Figure 2:**
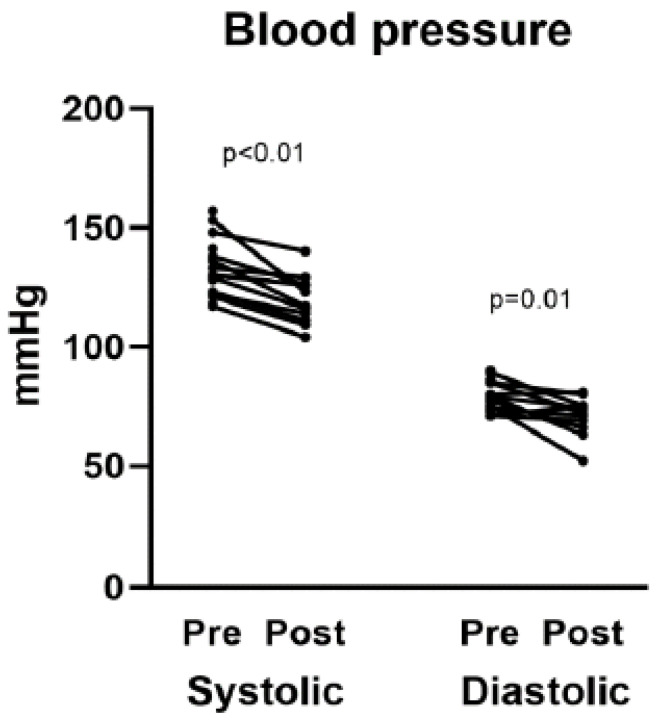
Differences in systolic and diastolic blood pressures pre- and post-finerenone treatment in patients with nondiabetic chronic kidney disease (n = 11).

**Figure 3:**
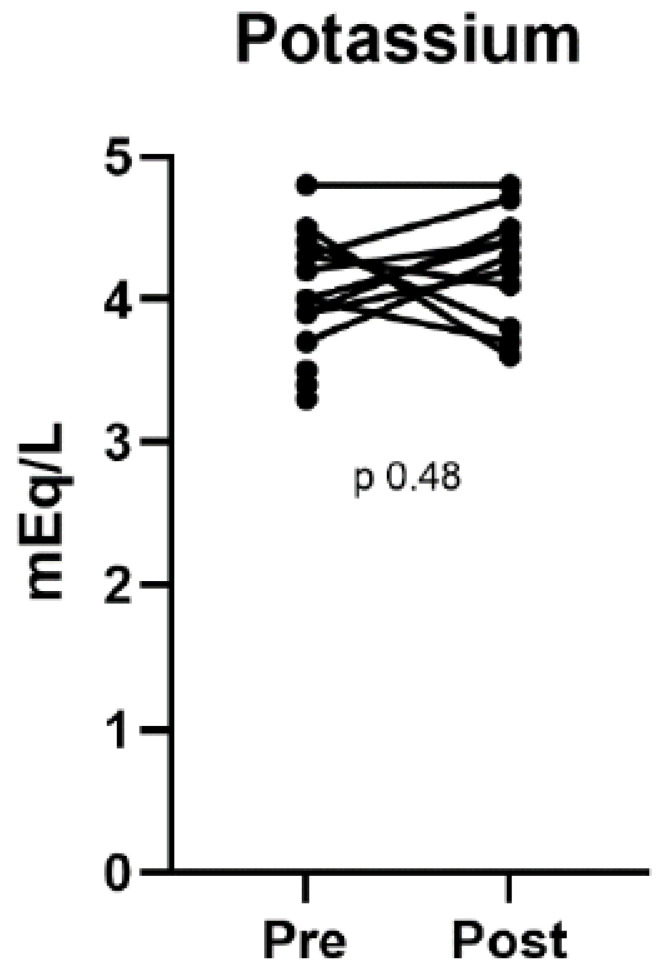
Changes in potassium levels pre- and post-finerenone treatment in patients with nondiabetic chronic kidney disease (n = 12).

Table 2 summarizes the treatment outcomes, including BP, UACR, urine protein-to-creatinine ratio (UPCR), and other laboratory values. Table 3 compares pre- and post-treatment BP and laboratory values, highlighting the statistically significant changes in BP, UACR, and UPCR. Following finerenone treatment, significant reductions in systolic and diastolic BP were observed: 117 mmHg (111–126 mmHg) for systolic BP (*p* < 0.01) and 72 mmHg (66–75 mmHg) for diastolic BP (*p* = 0.01). Albuminuria also improved significantly, as indicated by a decrease in the UACR from 90.0 to 39.3 mg/g (*p* = 0.04). The UPCR also decreased from 1.4 to 0.4 g/g (*p* < 0.01). Figure 1, Figure 2 and Figure 3 illustrate the differences in UPCR, UACR, and BP pre- and post-treatment, along with changes in potassium levels, demonstrating the overall effectiveness of finerenone in managing albuminuria and BP without significant adverse effects.

No significant changes were observed in the levels of potassium (4 mmol/L vs. 4.4 mmol/L, *p* = 0.48), albumin (43.5 g/L vs. 41.7 g/L, *p* = 0.31), or hemoglobin (14.5 g/dL vs. 13.9 g/dL, *p* = 0.08). Although a slight decline in eGFR from 66 to 58 mL/min/1.73 m^2^ (*p* < 0.01) was observed, this change was consistent with previous studies in patients with CKD using finerenone.

No adverse events, including hyperkalemia or hypotension, were reported during the treatment period. The results showed that finerenone effectively reduced albuminuria and BP in patients with nondiabetic CKD without causing significant adverse effects.

## 4. Discussion

Nondiabetic CKD can be classified into several subtypes depending on the underlying pathology. IgA nephropathy is the most prevalent form of nondiabetic CKD, accounting for approximately 52.68% of all cases [9,10]. The deposition of IgA antibodies in glomeruli is a defining characteristic, resulting in inflammation and injury. FSGS was the second most common pathology observed in this cohort. Scarring in particular regions of the renal filtration units is the major characteristic feature of FSGS and a primary contributor to nondiabetic CKD, which may result in progressive renal failure [11,12].

A recent study that identified significant clinical features and outcomes included over 500,000 patients with nondiabetic CKD. Ninety-four percent of patients were diagnosed with stage 3 CKD. The median age of the patients was 75 years, and 60.5% were female [13]. The baseline eGFR was within the range for patients with stage 2 CKD. The UACR is another important marker for assessing renal function and is frequently used to evaluate kidney health. A normal UACR is less than 30 mg/g, while microalbuminuria is indicated by values between 30 and 300 mg/g. Macroalbuminuria is indicated by values exceeding 300 mg/g [14,15]. An elevated UACR in individuals with nondiabetic CKD suggests the progression of renal disease and an increased risk of cardiovascular disease. The UACR in our patients clearly indicated progressive CKD.

RAAS inhibitors are the cornerstone of nondiabetic CKD management. SGLT2 inhibitors, including dapagliflozin and finerenone, have demonstrated substantial nephroprotective benefits in patients with CKD by delaying renal function decline and reducing albuminuria. These medications provide direct renal benefits independent of their impact on blood glucose levels [16,17].

Hyperkalemia, a significant adverse effect linked to finerenone, has been observed more frequently in individuals who were administered the drug during the FIDELIO-DKD clinical trial than in those who were administered a placebo. 21.4% of patients on finerenone developed moderate hyperkalemia, with serum potassium levels above 5.5 mmol/L. This starkly contrasts with 9.2% of the patients who received a placebo. Modest hyperkalemia, defined as serum potassium levels exceeding 6.0 mmol/L, was observed in 4.5% of the finerenone cohort. Conversely, this condition was observed in 1.4% of patients in the placebo cohort [18,19]. The percentage of treatment terminations, which may be attributed to hyperkalemia, was exceedingly low despite the elevated incidence [19]. The rate of finerenone use was only 2.3%, whereas the placebo rate was only 0.9%. In these investigations, no fatalities due to hyperkalemia were observed [19]. However, in the present study, none of the patients reported hyperkalemia. Potassium levels remained within the recommended range during the study period.

Finerenone has been shown to slightly reduce BP. Specifically, the mean systolic BP decreased by 2–4 mm Hg, and the diastolic BP decreased by 1–2 mm Hg during the first month of treatment. These effects persisted over time. A study assessing ambulatory BP found that finerenone significantly reduced 24 h systolic BP. Adjustments indicated that finerenone reduced systolic BP by 8.3 mmHg with a 10 mg dosage and 11.2 mmHg with a 15 mg dosage, compared to placebo [20].

In clinical trials, including FIDELIO-DKD and FIGARO-DKD, hypotension was observed in 4.6% and 3.0% of patients who received finerenone and placebo, respectively. Most hypotensive episodes were classified as mild to moderate and frequently resolved without medication cessation. The rate of hypotension-related hospitalizations was similar between the two groups, with only 0.1% of patients discontinuing finerenone owing to hypotension [20]. Similar to these findings, the blood pressure reduction reported in our cohort (≈14 mmHg systolic) is larger than in previous studies (2–4 mmHg), suggesting that the cohort is not comparable, and this should be discussed as a possible bias.

A recent study found that finerenone substantially reduced the UACR in individuals with diabetic kidney disease, suggesting its potential to lower albuminuria, a critical indicator of renal impairment [21]. A decrease in the UACR may also suggest a reduction in cardiovascular events in nondiabetic individuals and a delay in CKD [21]. Significant UACR improvement was observed in our patients, supporting the benefits of finerenone in nondiabetic CKD.

The median age of 38.5 years in our study population may not be representative of most nondiabetic CKD cohorts, as highlighted by other studies, where the median age is 75 years. This discrepancy should be considered when interpreting our findings [22].

In our cohort, eGFR declined slightly, which aligns with previous findings. The FIDELIO-DKD trial reported an initial median eGFR decrease of 3.3 mL/min/1.73 m^2^ in patients receiving finerenone after 4 months, compared to a drop of 0.8 mL/min/1.73 m^2^ in the placebo group. This initial decline, observed after discontinuation of the drug, was deemed reversible. The decline follows a power function associated with finerenone exposure, suggesting that, despite the initial reduction, long-term effects may stabilize or improve [23,24]. However, given the short duration of our study, it remains challenging to determine whether this decline represents a hemodynamic dip similar to that seen with RAAS inhibitors or a marker of CKD progression.

Nondiabetic chronic kidney disease (CKD) includes various underlying causes, such as glomerulonephritis and hypertension, which can affect treatment responses and outcomes. This study did not stratify results by specific CKD causes, making it difficult to tailor treatments for diverse patient populations.

Additionally, the small sample size (n = 16) and the absence of a comparator group limit the generalizability of the findings. Like many studies examining the effects of this drug on nondiabetic CKD over short follow-up periods, this exploratory study offers limited insights due to its retrospective design, which may introduce information biases and confounding factors. Notably, 90% of the cohort was on SGLT2 inhibitors, which can independently lower albuminuria and blood pressure. Subgroup analyses were not feasible with our current data. Finally, the study’s duration may be insufficient to assess long-term outcomes, such as kidney failure or cardiovascular events, which are essential for understanding the full impact of the treatment.

To conclude, finerenone shows promise in treating CKD, particularly among patients with diabetes. Our findings suggest that finerenone may reduce albuminuria and BP in nondiabetic CKD patients, warranting larger, controlled studies. This exploratory study provides preliminary data on its safety and efficacy in nondiabetic CKD, with no major adverse effects observed in a Saudi population, though initial eGFR decline was consistent with prior studies. Future studies should explore its long-term effects, comparative efficacy with other treatments, and potential benefits of combination therapies in diverse populations and nondiabetic CKD.

## Figures and Tables

**Table 1:** Baseline characteristics of patients with nondiabetic chronic kidney disease who received finerenone. BMI, body mass index; FSGS, focal segmental glomerulosclerosis; IgA, immunoglobulin A; BP, blood pressure; RAAS, renin–angiotensin–aldosterone system; SGLT2, sodium–glucose transport protein 2; HbA1C, hemoglobin A1c; eGFR, estimated glomerular filtration rate; UPCR, urine protein-to-creatinine ratio; UACR, urine albumin-to-creatinine ratio; LDL, low-density lipoprotein.

	Total (N = 16)
Demographics	
Age, years	38.5 (35–50)
Sex, male	9 (56.3%)
BMI	29.0 (27.2–30.0)
Primary renal disease	
IgA	8 (50%)
FSGS	3 (18.8%)
Others	5 (31.3%)
Blood pressure	
Systolic BP	131.5 (122.5–139.5)
Diastolic BP	79 (74.5–85)
RAAS inhibitors	16 (100%)
SGLT2 inhibitors	15 (93.8%)
Diuretics	3 (18.8%)
Statin	13 (81.3%)
Potassium binders	2 (12.5%)
Immunosuppressants	7 (43.8%)
▪ Mycophenolate mofetil	6 (37.5%)
▪ Hydroxychloroquine	1 (6.3%)
▪ Prednisolone	2 (12.5%)
Baseline laboratory results	
HbA1C (n = 14)	5.7 (5.4–6.0)
Creatinine	120 (90.5–153)
eGFR	66 (47–82.5)
UPCR (n = 15)	1.4 (0.9–2.2)
UACR (n = 12)	90.0 (58.8–132.5)
Potassium	4 (3.8–4.4)
Albumin	43.5 (38.8–45.0)
Hemoglobin	14.5 (12.7–15.6)
Total cholesterol	4.7 (3.9–6.5)
LDL	2.8 (2.2–4.1)
Finerenone treatment	
Dosage	10 (10–15)

**Table 2:** Overall treatment outcomes in patients with nondiabetic chronic kidney disease after finerenone therapy. BP, blood pressure; eGFR, estimated glomerular filtration rate; UPCR, urine protein-to-creatinine ratio; UACR, urine albumin-to-creatinine ratio; LDL, low-density lipoprotein.

Blood pressure (n = 11)	
Systolic BP	117 (111–126)
Diastolic BP	72 (66–75)
Adverse events	
Any adverse event	0
Post-treatment laboratory results	
Creatinine (n = 12)	119 (100.5–151)
eGFR (n = 12)	58 (46–76)
UPCR (n = 10)	0.4 (0.3–1.0)
UACR (n = 8)	39.3 (20.9–108.7)
Potassium (n = 12)	4.4 (4.0–4.5)
Albumin (n = 12)	41.7 (39.4–44.5)
Hemoglobin (n = 12)	13.9 (11.8–15.4)
Total cholesterol (n = 6)	4.9 (3.9–5.2)
LDL (n = 6)	2.5 (2.2–2.9)

**Table 3:** Comparison of blood pressure and laboratory results pre- and post-finerenone treatment in patients with nondiabetic chronic kidney disease. BP, blood pressure; eGFR, estimated glomerular filtration rate; UPCR, urine protein-to-creatinine ratio; UACR, urine albumin-to-creatinine ratio; LDL, low-density lipoprotein.

	Pre-Treatment	Post-Treatment	*p*-Value
Blood pressure (n = 11)			
Systolic BP	131.5 (122.5–139.5)	117 (111–126)	<0.01
Diastolic BP	79 (74.5–85)	72 (66–75)	0.01
Laboratory results			
Creatinine (n = 12)	120 (90.5–153)	119 (100.5–151)	<0.01
eGFR (n = 12)	66 (47–82.5)	58 (46–76)	<0.01
UPCR (n = 10)	1.4 (0.9–2.2)	0.4 (0.3–1.0)	<0.01
UACR (n = 8)	90.0 (58.8–132.5)	39.3 (20.9–108.7)	0.04
Potassium (n = 12)	4 (3.8–4.4)	4.4 (4.0–4.5)	0.48
Albumin (n = 12)	43.5 (38.8–45.0)	41.7 (39.4–44.5)	0.31
Hemoglobin (n = 12)	14.5 (12.7–15.6)	13.9 (11.8–15.4)	0.08
Total cholesterol (n = 6)	4.7 (3.9–6.5)	4.9 (3.9–5.2)	0.25
LDL (n = 6)	2.8 (2.2–4.1)	2.5 (2.2–2.9)	0.17

## Data Availability

The original contributions presented in this study are included in the article. Further inquiries can be directed to the corresponding author.
